# A genetically supported drug repurposing pipeline for diabetes treatment using electronic health records

**DOI:** 10.1016/j.ebiom.2023.104674

**Published:** 2023-07-01

**Authors:** Megan M. Shuey, Kyung Min Lee, Jacob Keaton, Nikhil K. Khankari, Joseph H. Breeyear, Venexia M. Walker, Donald R. Miller, Kent R. Heberer, Peter D. Reaven, Shoa L. Clarke, Jennifer Lee, Julie A. Lynch, Marijana Vujkovic, Todd L. Edwards

**Affiliations:** a Division of Genetic Medicine, Department of Medicine, Vanderbilt University Medical Center, Nashville, TN, USA; b Vanderbilt Genetics Institute, Vanderbilt University Medical Center, Nashville, TN, USA; c VA Informatics and Computer Infrastructure, VA Salt Lake City Health Care System, Salt Lake City, UT, USA; d Medical Genomics and Metabolic Genetics Branch, National Human Genome Research Institute, National Institutes of Health, Bethesda, MD, USA; e Division of Epidemiology, Department of Medicine, Vanderbilt University Medical Center, Nashville, TN, USA; f Nashville VA Medical Center, Nashville, TN, USA; g Medical Research Council, Integrative Epidemiology Unit, University of Bristol, Bristol, UK; h Bristol Medical School, UK; i Department of Surgery, University of Pennsylvania Perelman School of Medicine, Philadelphia, PA, USA; j Center for Healthcare Organization and Implementation Research, Bedford VA Healthcare System, Bedford, MA, USA; k Center for Population Health, Department of Biomedical and Nutritional Sciences, University of Massachusetts, Lowell, MA, USA; l VA Palo Alto Health Care System, Palo Alto, CA, USA; m Departments of Medicine and Endocrinology, Stanford University School of Medicine, Stanford, CA, USA; n Phoenix VA Health Care System, Phoenix, AZ, USA; o College of Medicine, University of Arizona, Phoenix, AZ, USA; p Departments of Medicine and Pediatrics, Stanford University School of Medicine, Stanford, CA, USA; q School of Medicine, University of Utah, Salt Lake City, UT, USA; r Corporal Michael J. Crescenz VA Medical Center, Philadelphia, PA, USA; s Department of Medicine, University of Pennsylvania Perelman School of Medicine, Philadelphia, PA, USA; t Department of Biostatistics, Epidemiology and Informatics, University of Pennsylvania Perelman School of Medicine, Philadelphia, PA, USA; u Population Health Sciences, University of Bristol, Bristol, UK

**Keywords:** Drug-repurposing, Mendelian randomization, Transcriptome-wide association study, Diabetes, Glucose, Hemoglobin A1c

## Abstract

**Background:**

The identification of new uses for existing drug therapies has the potential to identify treatments for comorbid conditions that have the added benefit of glycemic control while also providing a rapid, low-cost approach to drug (re)discovery.

**Methods:**

We developed and tested a genetically-informed drug-repurposing pipeline for diabetes management. This approach mapped genetically-predicted gene expression signals from the largest genome-wide association study for type 2 diabetes mellitus to drug targets using publicly available databases to identify drug–gene pairs. These drug–gene pairs were then validated using a two-step approach: 1) a self-controlled case-series (SCCS) using electronic health records from a discovery and replication population, and 2) Mendelian randomization (MR).

**Findings:**

After filtering on sample size, 20 candidate drug–gene pairs were validated and various medications demonstrated evidence of glycemic regulation including two anti-hypertensive classes: angiotensin-converting enzyme inhibitors as well as calcium channel blockers (CCBs). The CCBs demonstrated the strongest evidence of glycemic reduction in both validation approaches (SCCS HbA1c and glucose reduction: −0.11%, p = 0.01 and −0.85 mg/dL, p = 0.02, respectively; MR: OR = 0.84, 95% CI = 0.81, 0.87, p = 5.0 x 10–25).

**Interpretation:**

Our results support CCBs as a strong candidate medication for blood glucose reduction in addition to cardiovascular disease reduction. Further, these results support the adaptation of this approach for use in future drug-repurposing efforts for other conditions.

**Funding:**

10.13039/100000002National Institutes of Health, 10.13039/501100000265Medical Research Council Integrative Epidemiology Unit at the 10.13039/501100000883University of Bristol, 10.13039/501100000265UK Medical Research Council, 10.13039/100000968American Heart Association, and 10.13039/100000738Department of Veterans Affairs (VA) Informatics and Computing Infrastructure and VA Cooperative Studies Program.

**Research in context**

**Evidence before this study**

Medications with genetic support are significantly more likely to make it through clinical trials. Drug-repurposing is a relatively quick and inexpensive means for identification of new pharmacologic interventions for various diseases.

**Added value of this study**

This study demonstrates a successful implementation of a genetically-supported drug-repurposing pipeline for diabetes treatment. Further, this approach can be readily adapted and applied to other diseases and as such it has the potential to identify/prioritize drug repurposing targets for these conditions.

**Implications of all the available evidence**

Our results identified two anti-hypertensive medication classes, calcium channel blockers and angiotensin-converting enzyme inhibitors, as genetically supported drug-repurposing targets that demonstrated glycemic measurement reduction in real-world clinical populations. Further, these results suggest patients with diabetes or pre-diabetes could benefit from preferential use of these medication classes when they present with comorbid hypertension or other cardiovascular conditions.

## Introduction

An estimated 463 million individuals globally have a diabetes diagnosis^1^ and by 2045 this number is expected to reach 700 million. These rising case numbers are primarily due to type 2 diabetes (T2D) cases, the most prevalent form of the disease.^1^ Major comorbidities of T2D include heart disease, peripheral artery disease, stroke, eye disease, kidney disease, and neuropathy.^2^, ^3^, ^4^

Early stages of glycemic dysregulation can be effectively managed with behavioral modifications and metformin monotherapy. However, as T2D progresses, the concurrent use of multiple medications acting on distinct pathophysiologic pathways is often needed to control blood glucose levels and reduce development of complications as T2D progresses.^5^, ^6^, ^7^, ^8^ Regardless, a high proportion of patients with T2D demonstrate poor or inadequate glycemic control.^9^

The reason for poor glycemic control is multifactorial. These may include poor medication adherence due to undesired side-effects and costs,^10^^,^^11^ reduced treatment efficacy,^12^^,^^13^ and ineffective management of secondary metabolic and glucoregulatory dysfunction.^14^^,^^15^ Efforts to improve glycemic control include outreach to improve understanding of disease and development of new medications that target glucoregulatory dysfunctions to slow disease progression.^16^, ^17^, ^18^ T2D and comorbidities are also often treated separately, which may lead to increased morbidity and mortality due to polypharmacy.^19^

Drug repurposing provides an efficient, cost-effective means to increase therapeutic options by identifying medications that are currently approved for other indications for treatment of a disease.^20^ An example of this is acetylsalicylic acid, marketed as the analgesic Aspirin in 1899, which was subsequently discovered to both inhibit platelet aggregation^21^ and lower glucose.^22^ The advance of high-throughput technologies, such as genomics and transcriptomics, supports the development of new computational approaches for drug repurposing and identification of possible adverse drug events.^23^ These techniques leverage the highly druggable nature of human disease genes^24^ which may be implicated in diseases other than those indicated for derived medications.^25^, ^26^, ^27^

Serendipitous discoveries, usually a consequence of clinical observations of patients being treated for other conditions,^28^ have historically driven drug repurposing. Findings from real-world observations, however, are subject to biases, such as confounding by indication, reverse causality, and selective data missingness.^29^ Mendelian randomization (MR) may overcome some of the limitations of observational epidemiology.^29^ Here we propose a computational drug repurposing approach that identifies potential therapeutic candidates for T2D by 1) applying computational methods to genome-wide association study (GWAS) results, 2) evaluating these candidates through a observational self-controlled case series conducted in the electronic health records (EHR) using serendipitous clinical observations in the Department of Veterans Affairs (VA) Corporate Data Warehouse and Vanderbilt University Medical Center's synthetic derivative (VUMC SD), and 3) proxying the candidate's drug exposure as predicted expression of the therapeutic gene target using S-PrediXcan in a MR analysis.

## Methods

### Gene-based medication discovery

**Fig. 1:**
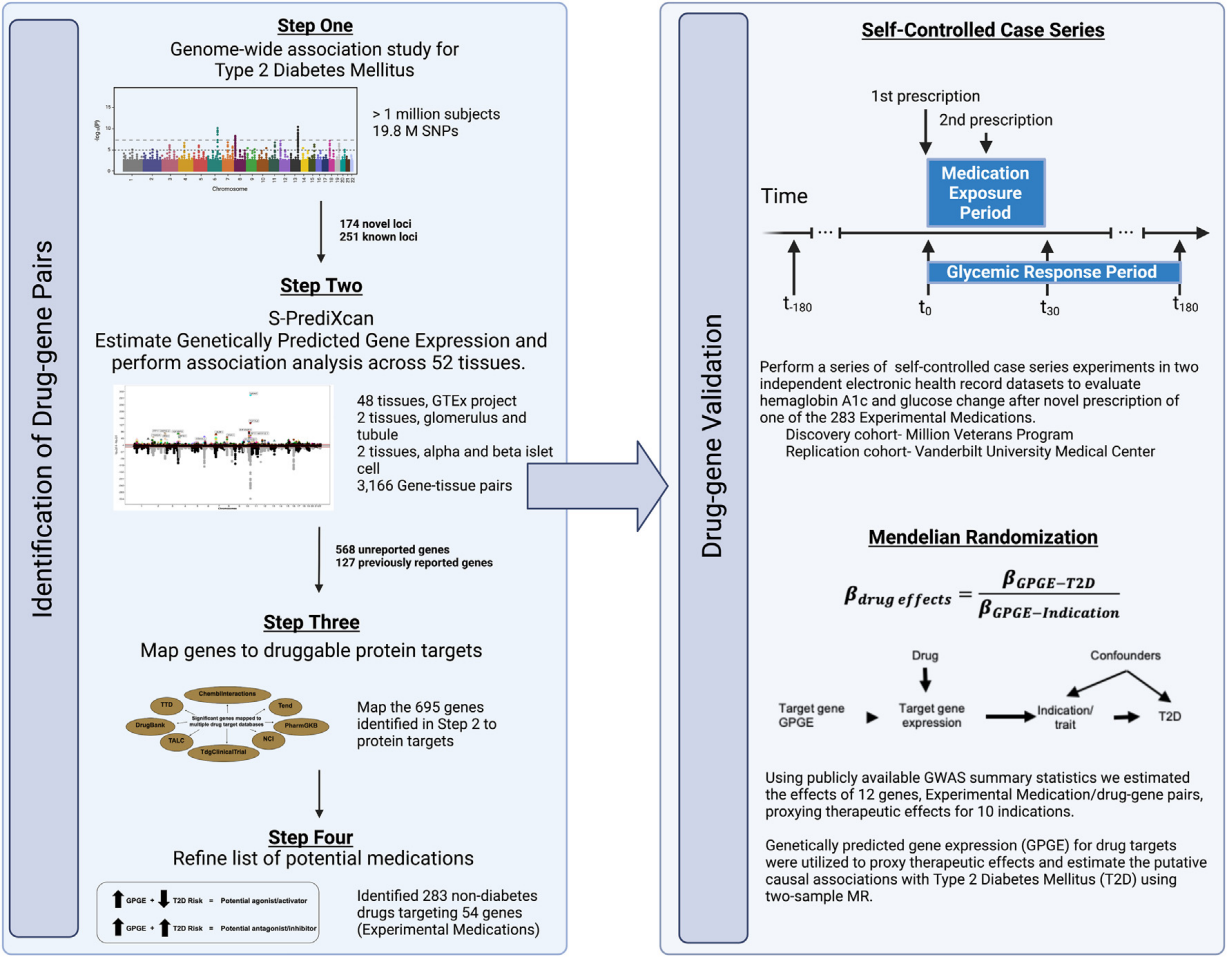
Study design. Steps 1–4 describe the process of identifying drug targets based on genetic evidence using a previously published genome-wide association study for Type 2 Diabetes Mellitus. Following identification of potential medications based on drug-gene pairing the results are validated using two approaches, self-controlled case series and mendelian randomization (MR). The general design of these two approaches are described briefly.

As described previously,^30^ we used summary statistics from the largest multi-ethnic GWAS of T2D, which included over 1.4 million participants, to estimate genetically-predicted gene expression (GPGE) of individual genes and performed a transcriptome-wide association study (TWAS) using s-PrediXcan. The results and associated methods have been described previously, however; we will briefly introduce the prior work. The T2D GWAS included 228,499 cases and 1,178,783 controls. The meta-analysis included summary statistics from 42 individual studies from across the globe including patients with African American, European, Hispanic, and South and East Asian ancestry. The imputation of GPGE was based on version 7 predictors for 52 tissues including 48 from GTEx, two kidney tissues (glomerulus and tubule),^31^ and two tissues from an alpha and beta islet cell reference^32^ and signals were refined using colocalization analyses.^30^ This analysis identified 695 significant genes (Reference 30, Supplemental Table S15). Next these genes were mapped to multiple drug targets (Reference 30, Supplemental Table S17, filtered on PP.H4. abf ≥ 0.8, for unique gene names) using publicly available databases: ChEMBL,^33^ Drug Gene Interaction Database (DGIdb),^34^ and National Cancer Institute Drug Dictionary.^35^ For this step we used these publicly available well-curated databases to identify genes that had a known association with a drug in at least one of these databases. Genes that were identified as having a known drug target by one of these databases were considered mapped to a drug and were included as a drug–gene pair for further evaluation. Further refinement included pairing the direction of GPGE and T2D risk with drug effects. The direction of effect for the GPGE association is based on the original s-PrediXcan analysis where effects are written with regard to a single standard deviation increase in the GPGE for the identified gene. This may result in an increase or decrease in T2D risk. For example, genes with increased GPGE that were associated with a decrease in T2D risk would need to map to a drug that acts as an activator or agonist of the associated protein or pathway. Conversely, drugs that act as an antagonist, inhibitor or blocker would correspond with a gene for which increasing GPGE associated with an increase in T2D risk. This process is visualized in Fig. 1, steps 1–4. Drug-gene pairs with correlated functions that were not identified previously for use in diabetes management were then included as experimental medications for the subsequent analyses to evaluate their potential for repurposing including a self-controlled case series and MR studies.

### Self-controlled case series data sources

Two separate EHR systems were identified as data sources for the self-controlled case series. The discovery population included clinical, prescription, and laboratory data from the Corporate Data Warehouse (CDW) of the Veterans Health Administration (VHA), a national US data repository that provides access to the EHRs of all individuals who received care in the VHA. All study variables were extracted from the CDW in April 2020 by an experienced programmer on Microsoft SQL Server via the VA Informatics and Computing Infrastructure (VINCI) computing environment.^36^

The replication site data were collected from the VUMC SD, a de-identified copy of the electronic medical record with Health Insurance Portability and Accountability Act of 1996 (HIPAA) identifiers removed.^37^ The SD contains clinical data on approximately 3.2 million individuals and includes basic demographics; text from clinical care notes; laboratory values; inpatient and outpatient medication data; International Classification of Disease (ICD) and Current Procedural Terminology (CPT) codes; and other diagnostic reports. Drug exposures were identified using previously described electronic-prescribing tools and MedEx.^38^ The utility of these methods for medication extraction from the EHR have been shown previously.^39^^,^^40^ All valid medication exposures required one of the following indications to be documented: route, frequency, dose, or duration. All study variables were extracted from the SD by an experienced programmer in January 2020.

### Self-controlled case series study design

**Fig. 2:**
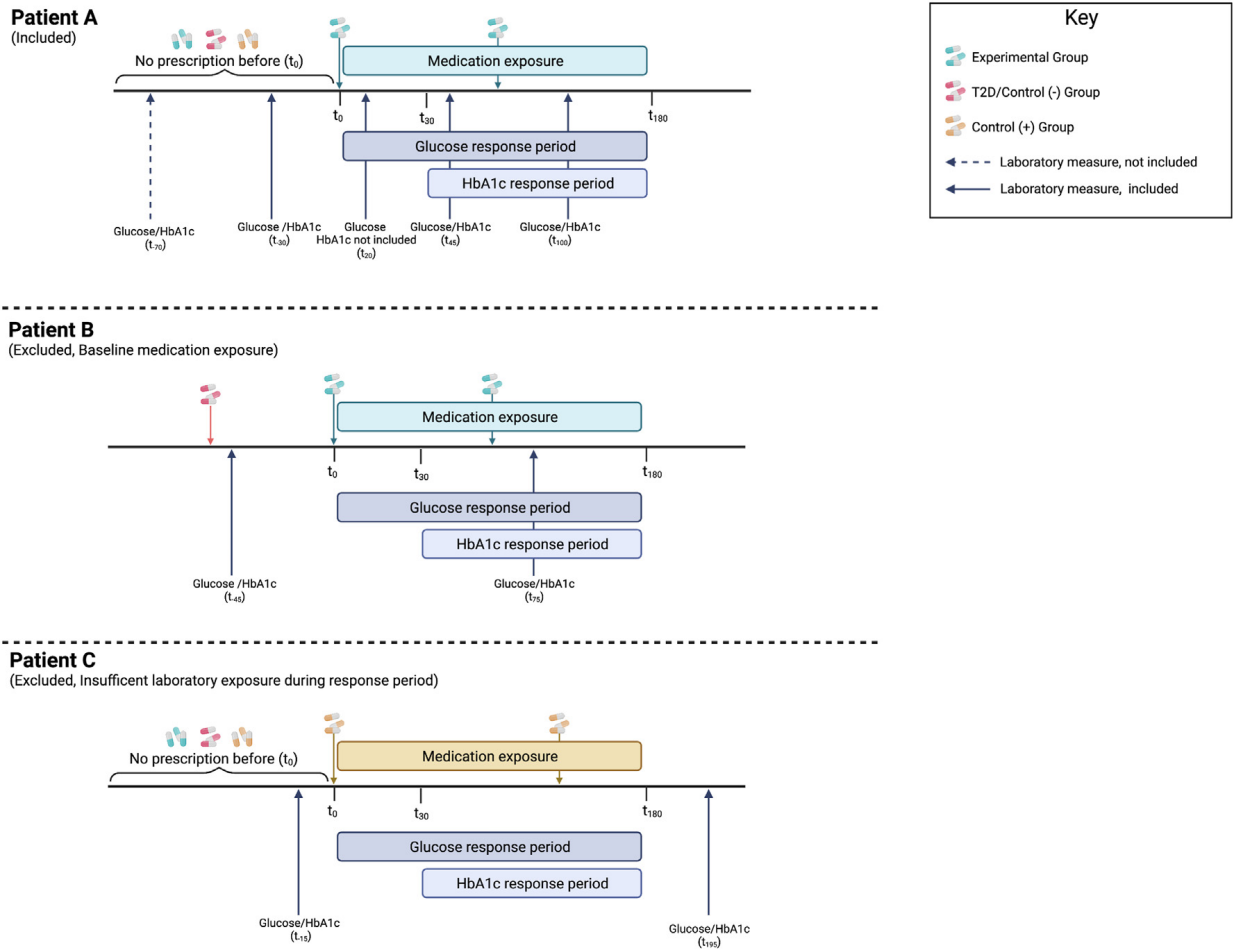
Examples of the medication exposure time period for the self-controlled case series for determination of patient inclusion or exclusion. Patient A demonstrates a patient that would be included in the study based on study design. Specifically, this patient was prescribed a medication belonging to the experimental group and had a subsequent mention of the medication in their records in the following 6 months. They had no documented exposure to a medication belonging to one of the other medication groups in the period preceding t0. They also had glucose and hemoglobin A1c (HbA1c) measures collected in the six months before medication exposure and during the response periods. Conversely, patients B and C were excluded from this study. Patient B represents patients that were excluded from the study because they had a medication exposure from another medication group prior to t0. Patient C is an example of a patient that would be excluded due to insufficient data. In this example, Patient C had no glucose and HbA1c measurements in the six-month exposure period. Likewise, patients without a glucose or HbA1c in the six months before t0 would also be excluded from the study due to insufficient laboratory exposure.

We utilized a self-controlled case series study design within the discovery and replication populations to evaluate the effects of initial medication use on hemoglobin A1c (HbA1c) and glucose. The design varied slightly between the two sites to accommodate variations in data availability and population. These variances are discussed below. The general design of this study is displayed in Fig. 2.

Medications evaluated in this series were grouped in to three sets: 1) experimental, the gene-based medication set described previously; 2) diabetes/control (−), medications that are prescribed for the treatment of T2DM; and 3) control (+), medications belonging to classes that had previously described increasing effects on glucose or HbA1c or were implicated in a previous MR study.^41^ The complete list of medications and their corresponding set are available in Supplemental Tables S1a and b. To ensure the effects on glucose or HbA1c were due to a specific medication, each individual medication series included patients that were prescribed the specific medication when the drug was initially prescribed, t_0_, but excluded patients prescribed a medication in one of the other groups ever before, simultaneously at t_0_, or in the six-month follow-up period.

Further, in the discovery set the VA patients were only included in a medication series if they received at least one subsequent refill of the given medication within 180 days of t_0,_ and had at least 90 days of cumulative exposure. Because records of a patient's prescription fill is less readily available in the VUMC SD, e.g. many patients will fill their prescriptions at a non-VUMC pharmacy so records of prescription pick-ups are absent in their record, this study required included subject EHRs in VUMC SD to have at least two mentions of the medication within 180 days of the initial drug mention, t_0_, to proxy prescription refill.

Finally, all patients included in the medication series were required to have both a baseline and follow-up HbA1c and glucose measured to evaluate response. HbA1c and glucose measures were restricted to outpatient measures and non-physiologic measures were excluded, e.g. HbA1c less than 3% or greater than 18% and glucose less than 5 mg/dL or greater than 2750 mg/dL. In the replication population we also excluded all laboratory measures that were collected within 9 months of a pregnancy ICD code or laboratory test (Supplemental Table S2). Because the discovery cohort is >90% male this exclusion was not applied.

The change in laboratory measure was defined as absolute change. The absolute change was calculated by taking the difference between the follow-up measure and the baseline measure. Baseline HbA1c was defined as the most recent measure in the six months prior to drug initiation. Follow-up HbA1c measure was defined as the first measure after 30 days and within the six months following drug initiation (Fig. 2). For glucose, we used the mean of all measures in the six months prior to drug initiation as the baseline value and the mean of all measures in the six months after drug initiation as the follow-up value.

### Statistical analysis

We assessed patient characteristics at drug initiation by comparing demographics, smoking status, body mass index, glycemic status, and Charlson Comorbidity Index categories across the three drug groups for both study populations. Using a self-controlled case series design, we performed pairwise t-test to determine statistically significant the changes in laboratory measures at the individual level. Drugs with a sample size less than 30 in the discovery population were not analysed to increase precision and reliability. We used SAS 9.2 (Cary, NC) for all data preparation and analysis in the discovery population and Rv3.2 for the replication population. We also performed a random-effects meta-analysis to estimate between-study and within medication class effect sizes for glucose and HbA1c change using STATA.

### Mendelian randomization

The MR analysis was structured around a subset of 12 genes identified as drug–gene pairs in Fig. 1, step 4. Two-sample MR was conducted by leveraging summary statistics from existing GWAS on ten different drug indications (angina,^42^ atrial fibrillation,^43^ bipolar disorder,^44^ coronary artery disease,^45^ congestive heart failure,^46^ epilepsy,^47^ glaucoma,^48^ pain,^49^ rheumatoid arthritis,^50^ systolic blood pressure^51^) in tandem with summary statistics from the largest T2D GWAS to date.^30^ For the 12 genes, the MR analysis utilized only tissues with statistically significant GPGE (p < 0.05) as the instrument and was conducted via the “TwoSampleMR” R package.^52^

We replicated a previously published approach using S-PrediXcan summary statistics as the instrumental variable in an MR analysis to proxy therapeutic targets.^41^ Tissue-specific GPGE summary statistics for each trait and T2D were combined to calculate the IVW MR association as follows:

$${\hat{\beta }}_{MR-IVW}=\frac{\sum_{i=1}^{t}\beta_{trait}\beta_{T2D}{\sigma_{\beta_{T2D}}}^{-2}}{\sum_{i=1}^{t}{\beta_{trait}}^{2}{\sigma_{\beta_{T2D}}}^{-2}}$$

$$se\left({\hat{\beta }}_{MR-IVW}\right)=\sqrt{\frac{1}{\sum_{i=1}^{t}{\beta_{trait}}^{2}{\sigma_{\beta_{T2D}}}^{-2}}}$$

where $\beta_{trait}$ represents GPGE per trait; and $\beta_{T2D}$ and $\sigma_{\beta_{T2D}}$ represent T2D GPGE and standard error, for *t* number of statistically significant GPGE tissues, respectively. Corresponding odds ratios (ORs) and 95% confidence intervals (95% CI) were calculated using ${\hat{\beta }}_{MR-IVW}$ and $se\left({\hat{\beta }}_{MR-IVW}\right)$.

The estimates (and 95% CIs) obtained from the MR analysis represent T2D risk per standard deviation change in gene expression. Furthermore, MR Egger regression was used to evaluate directional pleiotropy.^53^ Multivariable MR (MVMR) was conducted to estimate adjusted MR effects for indications that were correlated and shared therapeutic gene targets (e.g. ACE inhibitors are used to treat hypertension and congestive heart failure).^54^ We also conducted a negative control MR analysis to account for potential bias due to population stratification in our estimation of effects using a GWAS of natural hair color as the outcome.^55^^,^^56^

### Ethics

The studies obtained ethical approval from the affiliated organizations, Department of Veterans Affairs and Vanderbilt University Medical Center. For the VA discovery population, patient clinical data were analyzed as part of the Leveraging Electronic Health Information to Advance Precision Medicine research protocol, which has been approved by institutional review boards and research committees at 3 VA Medical Centers (Salt Lake City, Palo Alto, and West Haven) with approved waivers of informed consent and HIPAA authorization. For the VUMC SD patient data is de-identified and do not meet the 45 CFR 46 definition for human subjects research. As such, the study received non-human subjects determination from the associated institutional review board.

### Role of funders

The datasets, VUMC SD and MVP VA, used in the self-controlled case series were created by funding obtained from the funders. Funders were not involved in the study design, data analyses, interpretation, or writing of the results.

## Results

### Computational drug repurposing approach

We used a multi-step computational approach to drug repurposing that leveraged large scale GWAS and EHR data to identify and test potential non-diabetes medications for use in hemoglobin (HbA1c) and glucose control (Fig. 1). Steps 1–4 describe the process of identifying drug–gene pairs for evaluation and validation. The evaluation stage takes a two-pronged approach to evaluate these drug–gene pairs: a) a self-controlled case series to evaluate the response of HbA1c and glucose to initial medication exposure and b) a Mendelian randomization study to proxy therapeutic effects of a subset of the identified gene–drug pairs.

### Gene-based medication discovery (Fig. 1, steps 1–4)

Briefly, genes were identified in a previous large-scale GWAS and TWAS of diabetes risk.^30^ Publicly available databases of drug gene targets, indications, and interactions were consulted to identify drug–gene pairs. Drugs were selected such that the drug targeted a gene that was associated with diabetes risk via GPGE, the drug was not used in diabetes management, and where the action of the drug would be predicted to mitigate increases in diabetes risk.

Summary statistics for 19.8 million single nucleotide polymorphisms were utilized in S-PrediXcan models for GPGE estimation across 52 tissues. We identified 695 unique genes that were associated with diabetes risk at a p-value threshold of 1.92 x 10^−7^ [logistic regression], including: 568 genes for which a relationship was not previously reported and 127 with a known relationship to diabetes. We further refined the list of target drug–gene pairs by comparing medication and gene effects. For example, if a drug was an activator or agonist of the associated pathway or enzyme activity, we expect that increasing GPGE of the corresponding gene would be associated with decreased diabetes risk, and vice-versa for inhibitors. We identified 283 drugs with repurposing potential for diabetes that targeted 54 genes, 7.7% of the unique genes reported from the TWAS.

### Self-controlled case series

To test for the impact of initial medication start on HbA1c and glucose using EHRs, we designed a self-controlled case series to evaluate the change in these laboratory measures in the six months following medication initiation (Fig. 2). We restricted medication starts to a single experimental medication, defined by a gene–drug pair identified previously, with no preceding prescription of control medications (established medications known to raise or lower HbA1c and glucose). These medications were classified as glucose-reducing or glucose-increasing control medications based on prior knowledge of their effects on HbA1c or glucose. Overall, 68 medications were identified as possible control medications (Supplemental Table S1a and b). Further, we repeated this self-controlled case series for the glucose-reducing or glucose-increasing medications using identical restrictions on medication exposure and initial start to evaluate design performance (Fig. 2). We performed the self-controlled case series independently in two large EHR systems, the Veteran's Administration (VA) and Vanderbilt University Medical Center Synthetic Derivative (VUMC SD), separately for HbA1c and glucose.

#### Hemoglobin A1c

The VA discovery population for the HbA1c self-controlled case series included 124,357 patients: 40,780 (32.8%) in the T2D/glucose-reducing group, 25,170 (20.2%) in the glucose-increasing group, and 58,407 (47.0%) in the experimental group. The baseline characteristics of these patients are summarized in Supplemental Table S3a. The VUMC SD replication population was one-tenth the size of the VA population, including 15,365 patients: 4439 (28.9%) in the T2D/glucose-reducing group, 3912 (25.5%) in the glucose-increasing group, and 7014 (45.6%) in the experimental group. The baseline characteristics of these patients are summarized in Supplemental Table S3b.

The paired t-test results for HbA1c change in both the discovery and replication populations are summarized in Table 1. Analyzed medications were required to have at least 30 prescriptions in both sites. All T2D/glucose-reducing medications demonstrated substantial reductions in HbA1c in the discovery population with similar results in the replication set. The greatest reduction in HbA1c in the VA population was glyburide and glipizide, second-generation oral sulfonylureas, with mean changes of −1.3% and −1.1%, respectively (p < 0.001 [paired t-test]). The control (+) medications had more inconsistent effects on HbA1c across the two sites. Of the 16 glucose-increasing medications, only amitriptyline had evidence to support an increase in HbA1c in both populations. Seven additional medications were associated with increases in the discovery population but not in the replication. We observed consistent direction of effects with these 7 medications in the replication population but the substantially lower sample sizes potentially reduced the power to detect effects.

**Table 1:** HbA1c paired t-test results from VA LEAPS with replication at Vanderbilt (N ≥ 30 at both sites).

GROUP	DRUG	VA LEAPS overall					Vanderbilt overall				
		n	Mean Δ	95% C.I.		p	n	Mean Δ	95% C.I.		p
				Lower	Upper				Lower	Upper	
Glucose decreasing	GLYBURIDE	7639	−1.1525	−1.1979	−1.107	<0.0001	225	−0.7408889	−0.9785815	−0.5031962	<0.0001
Glucose decreasing	GLIPIZIDE	7564	−1.0307	−1.0741	−0.9873	<0.0001	429	−0.6013986	−0.7672611	−0.4355361	<0.0001
Glucose decreasing	METFORMIN	25,589	−0.9612	−0.983	−0.9393	<0.0001	1732	−0.776097	−0.8561348	−0.6960592	<0.0001
Glucose decreasing	LIRAGLUTIDE	112	−0.8491	−1.1336	−0.5646	<0.0001	34	−0.4264706	−0.89595328	0.04301211	0.0736
Glucose decreasing	GLIMEPIRIDE	109	−0.8179	−1.1233	−0.5125	<0.0001	302	−0.7006623	−0.878078	−0.5232465	<0.0001
Glucose decreasing	INSULIN	8614	−0.7853	−0.8265	−0.7441	<0.0001	1558	−0.7970475	−0.8950016	−0.6990934	<0.0001
Glucose decreasing	PIOGLITAZONE	498	−0.4414	−0.5679	−0.3149	<0.0001	30	−0.24	−0.53962488	0.05962488	0.1122
Glucose decreasing	SITAGLIPTIN	103	−0.0777	−0.2962	0.1409	0.4825	35	−0.4257143	−0.9284599	0.0770313	0.0944
Experimental	LISINOPRIL	17,374	−0.0346	−0.0455	−0.0237	<0.0001	1145	−0.2383406	−0.3156355	−0.1610457	<0.0001
Experimental	BENAZEPRIL	579	−0.0813	−0.1321	−0.0306	0.0017	68	−0.3191176	−0.62066544	−0.01756986	0.0384
Experimental	PRAVASTATIN	3944	−0.0253	−0.0447	−0.00592	0.0105	284	−0.251669	−0.40619831	−0.09713972	0.0015
Experimental	VERAPAMIL	522	−0.0665	−0.1231	−0.0099	0.0214	81	−0.2185185	−0.4354824	−0.00155464	0.0484
Experimental	ISOSORBIDE DINITRATE	157	0.1231	0.0089	0.2372	0.0348	177	−0.01299435	−0.1621161	0.1361274	0.8637
Experimental	ENALAPRIL	577	−0.0426	−0.0898	0.00461	0.0768	123	0.06910569	−0.1325936	0.270805	0.4989
Experimental	ROSUVASTATIN	2149	0.0277	−0.00453	0.0599	0.0920	285	−0.08736842	−0.20676963	0.03203279	0.1509
Experimental	ISOSORBIDE	357	0.0604	−0.0232	0.1441	0.1563	171	−0.01169591	−0.1657714	0.1423795	0.8811
Experimental	ASPIRIN	3928	−0.0174	−0.0431	0.00824	0.1833	1834	−0.1676521	−0.2182151	−0.1170891	<0.0001
Experimental	TOPIRAMATE	739	−0.0242	−0.0612	0.0128	0.1992	53	−0.3113208	−0.70960458	0.08696307	0.1228
Experimental	LOVASTATIN	1844	−0.0153	−0.0436	0.013	0.2880	172	−0.2290698	−0.38846987	−0.06966967	0.0051
Experimental	SILDENAFIL	8563	−0.00697	−0.0215	0.00759	0.3479	51	−0.6882353	−1.2261789	−0.1502917	0.0132
Experimental	OXCARBAZEPINE	52	0.0404	−0.0603	0.141	0.4242	39	−0.2923077	0.7680527	0.1834373	0.2212
Experimental	LAMOTRIGINE	402	0.0247	−0.0386	0.088	0.4434	46	−0.273913	−0.7942324	0.2464063	0.2947
Experimental	PRILOCAINE	74	0.0514	−0.0881	0.1908	0.4655	127	0.0247244	−0.1739227	0.2148676	0.8352
Experimental	LEVETIRACETAM	193	0.0254	−0.0456	0.0964	0.4817	75	−0.02666667	−0.3173848	0.2640514	0.8555
Experimental	LIDOCAINE	331	0.0173	−0.0351	0.0697	0.5160	567	−0.1074074	−0.19241439	−0.02240043	0.0134
Experimental	SIMVASTATIN	21,353	0.00255	−0.00649	0.0116	0.5802	561	−0.142246	−0.28449198	−0.03422388	0.0099
Experimental	ATORVASTATIN	2328	0.00881	−0.0294	0.047	0.6512	619	−0.1924071	−0.2966929	−0.0881213	0.0003
Experimental	DICLOFENAC	2012	−0.00398	−0.0307	0.0228	0.7703	238	−0.04453782	−0.17240455	0.08332892	0.4933
Experimental	RAMIPRIL	102	0.0167	−0.1017	0.135	0.7806	96	−0.146875	−0.35919937	0.06544937	0.1729
Experimental	PREGABALIN	322	0.0027	−0.0835	0.0889	0.9508	36	−0.05277778	−0.4503729	0.3448173	0.7891
Glucose increasing	HYDROCHLOROTHIAZIDE	6016	0.1028	0.085	0.1205	<0.0001	664	0.05406627	−0.01880839	0.12694092	0.1457
Glucose increasing	PROPRANOLOL	1088	0.1415	0.0921	0.1909	<0.0001	94	0.09893617	−0.08261529	0.28048763	0.2820
Glucose increasing	PREDNISONE	829	0.2814	0.2088	0.3539	<0.0001	412	0.008009709	−0.1028593	0.1188787	0.8871
Glucose increasing	NORTRIPTYLINE	499	0.1074	0.0428	0.1721	0.0012	32	0.046875	−0.2186954	0.3124454	0.7213
Glucose increasing	METOPROLOL	3259	0.0385	0.014	0.063	0.0021	520	−0.02230769	−0.13145947	0.08684408	0.6882
Glucose increasing	ATENOLOL	2873	0.0401	0.0136	0.0666	0.0030	240	0.1520833	−0.006750816	0.310917483	0.0605
Glucose increasing	QUETIAPINE	1248	0.0493	0.0148	0.0839	0.0052	36	0.02222222	−0.2910418	0.3354863	0.8863
Glucose increasing	AMITRIPTYLINE	1269	0.0523	0.00909	0.0956	0.0177	141	0.1815603	0.02675168	0.33636889	0.0219
Glucose increasing	CITALOPRAM	3754	−0.0237	−0.0447	−0.00264	0.0274	339	−0.007079646	−0.1371858	0.1230265	0.9148
Glucose increasing	OLANZAPINE	292	0.104	−0.00161	0.2096	0.0536	54	0.011111	−0.1708453	0.1930675	0.9030
Glucose increasing	FLUOXETINE	2318	−0.0207	−0.047	0.00566	0.1239	176	0.009659091	−0.1351066	0.1544248	0.8954
Glucose increasing	PAROXETINE	1000	0.0381	−0.0112	0.0874	0.1297	55	−0.009090909	−0.2567106	0.2385288	0.9416
Glucose increasing	CARVEDILOL	655	0.0371	−0.0271	0.1014	0.2565	247	0.1809717	0.0079371	0.3540062	0.0405
Glucose increasing	CIPROFLOXACIN	54	−0.1167	−0.3366	0.1033	0.2922	182	0.1961538	0.01111352	0.38119418	0.0379
Glucose increasing	RISPERIDONE	643	0.0227	−0.0293	0.0747	0.3914	47	−0.3808511	−0.8242209	0.0625188	0.0905
Glucose increasing	EZETIMIBE	194	−0.0299	−0.1196	0.0598	0.5117	80	0.1925	−0.1369853	0.5219853	0.2484
Glucose increasing	SERTRALINE	5519	0.00351	−0.014	0.021	0.6937	151	−0.1264901	−0.29593777	0.04295764	0.1423
Glucose increasing	HYDROCORTISONE	54	0.0278	−0.1555	0.2111	0.7624	58	0.2448276	−0.1630528	0.652708	0.2343

Of the initial 283 experimental medications identified by drug–gene pairs, only 20 (7.1%) had sufficient data for inclusion in the HbA1c self-controlled case series. This set included four angiotensin-converting enzyme (ACE) inhibitors. Two demonstrated substantial reductions in HbA1c in the discovery and replication populations, and one, enalapril, had evidence for a reduction in the discovery but not the replication set. Ramipril did not consistently reduce HbA1c in either set. Lisinopril demonstrated the largest reduction (p < 0.001, mean change VA = −0.03% and VUMC = −0.24% [paired t-test]) in both populations. The calcium channel blocker verapamil also demonstrated reductions in HbA1c in both sets. Most statins had inconsistent effects. Pravastatin demonstrated a decrease in HbA1c in both populations.

We performed secondary analyses stratified by EHR-reported race in the discovery population to evaluate impacts on response due to population variation. Unfortunately, due to power concerns, these analyses could not be repeated for EHR-reported sex as the discovery population is >90% male and the replication site had too few instances across the majority of analyzed medications. These results are summarized in Supplemental Table S4. Consistent with the results from the total population analysis, lisinopril demonstrated a reduction in HbA1c across stratified groups, however, the mean reduction was slightly higher in patients with EHR-reported Black or Unknown race. It is important to note, that the four ACE inhibitors did not demonstrate a consistent trend as enalapril and verapamil both demonstrated greater reduction in the EHR-reported White than Black populations. We also observed a consistent inconsistency in response to statins.

#### Glucose

The VA discovery population for the glucose self-controlled case series included 678,501 patients: 51,645 (7.6%) in the T2D/glucose-reducing group, 235,493 (34.7%) in the glucose-increasing group, and 391,363 (57.7%) in the experimental group. The baseline characteristics of these patients are summarized in Supplemental Table S5a. The VUMC SD replication population was about one-tenth of the size of the VA population, including 67,155 patients: 11,889 (17.7%) in the T2D/glucose-reducing group, 9371 (14.0%) in the glucose-increasing group, and 45,895 (68.3%) in the experimental group. The baseline characteristics of these patients are summarized in Supplemental Table S5b.

The paired t-test results for glucose change in both the discovery and replication populations are summarized in Table 2. Consistent with the HbA1c results, glyburide and glipizide had the most substantial decrease in glucose (p < 0.001 [paired t-test]) in the discovery set. For the most part, all T2D/glucose-reducing medications demonstrated decreases in glucose. Some medications, such as sitagliptin, decreased glucose in the replication but not the discovery set (VA, p = 0.44 and VUMC, p = 0.002 [paired t-test for both]). These results suggest high variability in random glucose measurements may decrease statistical precision. The glucose-increasing medications demonstrated similar variability in results with dexamethasone exhibiting the only consistent increase in glucose across both populations (p < 0.001 [paired t-test]).

**Table 2:** Glucose paired t-test results from VA LEAPS with replication at Vanderbilt (N ≥ 30 at both sites).

GROUP	DRUG	VA LEAPS overall					Vanderbilt overall				
		n	Mean Δ	95% C.I.		p	n	Mean Δ	95% C.I.		p
				Lower	Upper				Lower	Upper	
Glucose decreasing	GLYBURIDE	8345	−43.1949	−45.2227	−41.1672	<0.0001	651	−6.447095	−15.00825	2.11406	1.3970E-01
Glucose decreasing	GLIPIZIDE	7161	−34.9926	−37.0172	−32.968	<0.0001	1117	−9.768414	−15.729241	3.807587	<0.0001
Glucose decreasing	METFORMIN	22,601	−31.7508	−32.7881	−30.7135	<0.0001	4381	−14.96459	−17.66529	−12.26389	<0.0001
Glucose decreasing	INSULIN	7786	−28.6124	−31.0302	−26.1945	<0.0001	4575	−5.448993	−8.83992	−2.058065	0.0016
Glucose decreasing	PIOGLITAZONE	404	−14.5794	−20.8713	−8.2876	<0.0001	100	−9.562057	−26.886532	7.762418	0.2761
Glucose decreasing	LIRAGLUTIDE	84	−28.9598	−46.5825	−11.3372	0.0016	52	−12.48878	−37.15229	12.17472	0.3142
Glucose decreasing	GLIMEPIRIDE	85	−23.4793	−39.7981	−7.1605	0.0053	703	−17.82565	−25.971571	−9.679738	<0.0001
Glucose decreasing	SITAGLIPTIN	85	−9.3883	−22.9627	4.1862	0.1727	77	−26.36202	−42.86386	−9.86019	0.0021
Experimental	SIMVASTATIN	136,659	0.4508	0.3568	0.5448	<0.0001	3498	0.734901	−1.813262	3.283064	0.5718
Experimental	FOSINOPRIL	12,212	−0.8226	−1.2394	−0.4059	0.0001	73	1.121298	−12.3272	14.5698	0.8685
Experimental	ROSUVASTATIN	6573	0.9923	0.4779	1.5066	0.0002	1411	2.51068	−1.169198	6.190559	0.1810
Experimental	LISINOPRIL	97,335	−0.2488	−0.3842	−0.1134	0.0003	6616	1.067576	−0.902239	3.037391	0.9020
Experimental	PRAVASTATIN	14,954	0.5336	0.2456	0.8215	0.0003	1660	3.462117	−0.7322978	7.6565323	0.1056
Experimental	VERAPAMIL	4583	−0.9979	−1.6193	−0.3764	0.0017	715	−2.061128	−6.49482	2.372563	0.3617
Experimental	TOPIRAMATE	4032	0.8667	0.3142	1.4193	0.0021	405	1.433802	−4.608238	7.475842	0.6411
Experimental	SILDENAFIL	38,485	0.3159	0.0942	0.5375	0.0052	461	6.203665	−1.884591	14.29192	0.1324
Experimental	ATORVASTATIN	5771	0.8247	0.1841	1.4652	0.0116	2829	−1.437549	−4.506864	1.631767	0.3585
Experimental	ASPIRIN	29,834	−0.297	−0.5489	−0.0452	0.0208	11,303	−0.3833803	−0.9497342	1.7164947	0.5730
Experimental	BENAZEPRIL	2616	−0.8457	−1.7148	0.0233	0.0565	368	1.318718	−5.138851	7.776286	0.6882
Experimental	OXCARBAZEPINE	329	2.2351	−0.0953	4.5654	0.0601	376	−12.84378	−22.936732	−2.750834	0.0128
Experimental	ENALAPRIL	2989	−0.6762	−1.4181	0.0656	0.0740	922	1.31505	−2.504694	5.134793	0.4994
Experimental	LOVASTATIN	14,595	0.2771	−0.0312	0.5854	0.0781	770	3.966773	−1.025044	8.958589	0.1192
Experimental	PREGABALIN	1220	1.1228	−0.1441	2.3898	0.0823	400	−4.517374	−13.390553	4.355804	0.3175
Experimental	DABIGATRAN ETEXILATE	831	1.2539	−0.4717	2.9795	0.1542	52	2.301677	−9.122465	13.725818	0.6876
Experimental	LEVETIRACETAM	1410	0.6273	−0.3889	1.6435	0.2261	947	−4.81774	−10.3211268	0.6856466	0.0861
Experimental	DOFETILIDE	165	1.7798	−1.2467	4.8063	0.2473	52	−3.194277	−17.53305	11.14449	0.6566
Experimental	DIPYRIDAMOLE	1326	−0.7342	−1.9806	0.5121	0.2480	70	5.131848	−3.252576	13.516272	0.2262
Experimental	SALMON CALCITONIN	723	0.8165	−0.615	2.2481	0.2632	72	−0.4615575	−11.11558	10.19246	0.9314
Experimental	LAMOTRIGINE	2340	0.4208	−0.343	1.1845	0.2801	467	2.711233	−3.841852	9.264317	0.4166
Experimental	ISOSORBIDE	1259	0.7299	−0.8707	2.3304	0.3711	1250	0.5219031	−3.957712	5.001518	0.8192
Experimental	PROPAFENONE	296	0.8757	−1.3405	3.092	0.4374	109	−2.98408	−11.335945	5.367785	0.4803
Experimental	SULINDAC	2637	0.255	−0.4727	0.9827	0.4920	64	−0.8106771	−19.34646	17.7251	0.9306
Experimental	PRILOCAINE	308	−0.8119	−3.2941	1.6703	0.5203	746	1.971907	−2.234013	6.177827	0.3577
Experimental	FLUVASTATIN	1802	0.2088	−0.4771	0.8946	0.5506	39	1.08315	−14.09033	16.25663	0.8859
Experimental	PENTOXIFYLLINE	805	−0.3254	−1.9554	1.3046	0.6953	105	6.625918	−12.57929	25.83113	0.4954
Experimental	DICLOFENAC	10,685	−0.0725	−0.4498	0.3048	0.7065	1328	0.446702	−3.445042	4.338445	0.8219
Experimental	RAMIPRIL	526	0.399	−1.7078	2.5057	0.7100	482	−1.003529	−6.855517	4.848459686	0.7363
Experimental	MICONAZOLE	1803	0.1451	−0.8235	1.1136	0.7690	80	−6.841524	−17.091672	3.408624	0.1878
Experimental	PHENYTOIN	2682	−0.0855	−0.9099	0.7389	0.8388	254	2.698407	−3.767747	9.164561	0.4119
Experimental	ISOSORBIDE DINITRATE	1469	0.1244	−1.1109	1.3597	0.8434	1256	−0.2453867	−4.74463	4.253856	0.9148
Experimental	LIDOCAINE	1841	0.093	−0.8702	1.0561	0.8499	6143	1.984721	0.1399591	3.8294838	0.0350
Experimental	MINOXIDIL	218	−0.3757	−4.649	3.8975	0.8626	415	−1.185078	−9.736265	7.36611	0.7855
Experimental	PRIMIDONE	882	−0.1221	−1.5594	1.3151	0.8676	125	−1.1066	−11.644133	9.430933	0.8357
Glucose increasing	ATENOLOL	25,545	0.5676	0.3127	0.8225	<0.0001	568	1.460915	−3.094773	6.016604	0.5290
Glucose increasing	PROPRANOLOL	8806	0.9974	0.5079	1.487	<0.0001	171	1.122807	−9.919777	12.165391	0.8412
Glucose increasing	RISPERIDONE	5015	1.2782	0.6408	1.9156	<0.0001	52	−20.94231	−55.2099	13.32529	0.2255
Glucose increasing	CARVEDILOL	2540	2.0708	1.054	3.0876	<0.0001	805	4.463354	−2.046625	10.973333	0.1787
Glucose increasing	HYDROCHLOROTHIAZIDE	55,715	2.1662	2.0019	2.3305	<0.0001	1731	0.5670133	−1.625194	2.759221	0.6120
Glucose increasing	PREDNISONE	10,064	3.3271	2.8554	3.7988	<0.0001	1058	1.359641	−2.993715	5.712997	0.5401
Glucose increasing	DEXAMETHASONE	814	11.0592	8.9244	13.1941	<0.0001	804	4.72236	1.306546	8.138174	0.0068
Glucose increasing	OLANZAPINE	2855	1.8426	0.906	2.7792	0.0001	58	−4.568966	−13.350344	4.212413	0.3019
Glucose increasing	AMITRIPTYLINE	10,722	0.7722	0.3477	1.1967	0.0004	204	−2.529412	−10.320404	5.261581	0.5228
Glucose increasing	METOPROLOL	20,349	0.5649	0.2479	0.8819	0.0005	1726	4.256833	1.197392	7.316273	<0.0001
Glucose increasing	NORTRIPTYLINE	3569	0.9445	0.2535	1.6356	0.0074	56	−7.928571	−27.27985	11.42271	0.4151
Glucose increasing	METHYLPREDNISOLONE	329	3.8446	0.8012	6.888	0.0135	35	20.8881	1.422146	40.354045	0.0005
Glucose increasing	HYDROCORTISONE	674	1.778	0.3158	3.2402	0.0172	97	8.06701	−0.3042293	16.43825	0.0588
Glucose increasing	QUETIAPINE	7939	0.5904	0.0905	1.0903	0.0206	30	−13.93333	−45.70653	17.83987	0.3772
Glucose increasing	NADOLOL	383	2.4766	0.2753	4.678	0.0276	59	−9.339266	−26.89005	8.21152	0.2912
Glucose increasing	BISOPROLOL	130	3.742	−0.1533	7.6373	0.0596	33	−2.742424	−20.8768	15.39195	0.7600
Glucose increasing	PAROXETINE	7083	0.5042	−0.0618	1.0702	0.0808	108	1.62037	−8.661824	11.902565	0.7553
Glucose increasing	LEVOFLOXACIN	420	−1.6014	−3.4438	0.241	0.0883	202	2.534653	−6.477816	11.547123	0.5798
Glucose increasing	DOXEPIN	1881	0.7812	−0.3126	1.8751	0.1615	36	5.777778	−14.16079	25.71634	0.5601
Glucose increasing	CIPROFLOXACIN	800	0.7613	−0.8172	2.3397	0.3441	142	10.5979	−7.865984	29.061789	0.2584
Glucose increasing	SERTRALINE	32,238	0.1053	−0.1203	0.3309	0.3602	248	4.165323	4.117053	12.447698	0.3229
Glucose increasing	EZETIMIBE	954	−0.52	−1.7218	0.6818	0.3961	145	6.453448	−3.820649	16.727546	0.2164
Glucose increasing	CITALOPRAM	30,433	−0.0787	−0.3139	0.1565	0.5118	389	−3.589974	−9.485146	2.305198	0.2319
Glucose increasing	FLUOXETINE	15,757	0.0989	−0.2237	0.4214	0.5480	207	−1.070048	−8.646338	6.506242	0.7809
Glucose increasing	SOTALOL	723	0.4891	−1.1468	2.1251	0.5574	55	−2.490909	−14.862197	9.880378	0.6880
Glucose increasing	MOXIFLOXACIN	126	−0.6569	−4.3452	3.0313	0.7250	140	−0.2695035	−9.122082	8.583075	0.9521

#### Consistency between glucose and HbA1c findings

Compared with HbA1c, 35 (12.4%) of the 283 medications identified by drug–gene pairs were included in the glucose analysis. Only one of these medications, oxcarbazepine, an anti-epileptic, had evidence for an effect in both sets. However, the direction of glucose change was inconsistent with glucose increasing in the VA discovery population and decreasing in the VUMC replication. None of the ACE inhibitors demonstrated a consistent impact on glucose in either population. In line with the HbA1c results, verapamil was associated with a consistent decrease in glucose in the VA (−0.82 mg/dL, p = 0.03 [paired t-test]). In the VUMC population, the point estimate indicated that verapamil decreased glucose but the wide confidence interval included the null, potentially due to power limitations of this smaller sample set. There were also inconsistent results for statin medications. In the discovery set, rosuvastatin increased glucose but the remaining statins including simvastatin, atorvastatin, pravastatin, lovastatin, and Fluvastatin, decreased glucose.

#### Meta-analysis

**Fig. 3:**
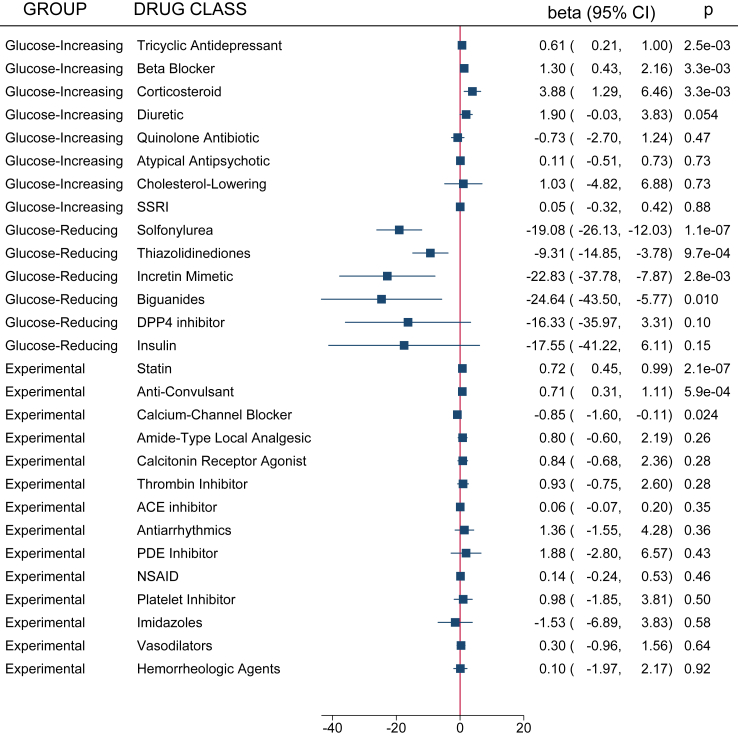
Medication group meta-analysis results from the self-controlled case series for hemoglobin A1c. All medications included in the analysis series were grouped by medication class and meta-analyzed. A forest plot of these results for hemoglobin A1c from the self-controlled case series in the discovery and replication datasets is presented. Whether they belonged to the control, glucose-decreasing or glucose-increasing, or experimental group is noted on the left-hand side of the figure.

**Fig. 4:**
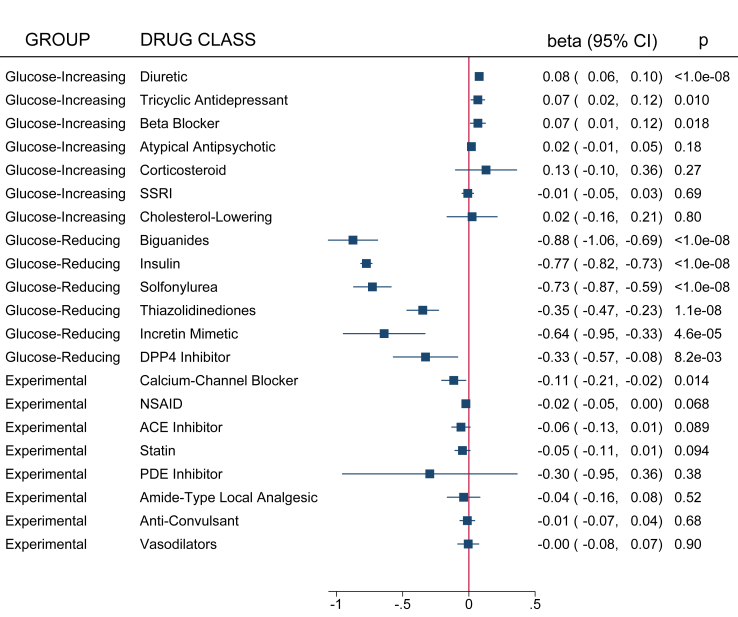
Medication group meta-analysis results from the self-controlled case series for glucose. All medications included in the analysis series were grouped by medication class and meta-analyzed. A forest plot of these results for glucose from the self-controlled case series in the discovery and replication datasets is presented. Whether they belonged to the control, glucose-decreasing or glucose-increasing, or experimental group is noted on the left-hand side of the figure.

We next performed a cross-site meta-analysis for both HbA1c and glucose in a self-controlled case series to estimate the combined effect for the given medications. The results of these analyses are available in Fig. 3, Fig. 4. There was evidence to support all T2D/glucose-reducing medications reducing HbA1c with effect sizes ranging from −0.33% for sitagliptin to −1.06% for glyburide (p < 0.001 for all [random effects meta-analysis]). For glucose, however, only half the T2D/control (−) medications were associated with a mean decrease in glucose. Four (26.7%) of the 15 glucose-increasing medications were associated with an increase in HbA1c as expected, with the most substantial increases being for propranolol and hydrochlorothiazide, respectively (0.12%, p = 1.0e^−5^; and 0.08%, p = 2.8 x 10^−14^ [random effects meta-analysis]). Both propranolol and hydrochlorothiazide also demonstrated increases in glucose. Interestingly, there was also some evidence that citalopram and fluoxetine decreased HbA1c (−0.02%, p = 0.04; and −0.03%, p = 0.01 [random effects meta-analysis]). However, evidence for their impact on glucose was limited. Corticosteroids had the most substantial impact on glucose. Dexamethasone and prednisone consistently increased glucose (7.87 mg/dL, p = 9.4 x 10^−3^; 1.32 mg/dL, p < 1.3 x 10^−16^ [random effects meta-analysis]). In the experimental group, only verapamil demonstrated a significant effect on HbA1c (−0.11%, p = 0.01 [paired t-test]). This effect was also observed in the glucose analysis (−0.85 mg/dL, p = 0.024). Three statins, simvastatin, rosuvastatin, and fluvastatin, had evidence for changes in glucose; however, the directions of effect varied among the medications.

To characterize the effect of medication group on HbA1c and glucose, we also performed a drug-class grouped meta-analysis (Fig. 3, Fig. 4, respectively). As expected, all six of the T2D/glucose-reducing medication groups lowered HbA1c with the most substantial decrease belonging to biguanides (−0.88%, p < 1.6 x 10^−13^ [paired t-test]). We saw similar magnitudes of reductions in glucose for DPP4 inhibitors and insulin (p = 0.10 and p = 0.15, respectively [paired t-test]). Three of the glucose-increasing medication groups, diuretics, tricyclic antidepressants, and beta blockers, demonstrated increases in HbA1c and glucose; while corticosteroids only demonstrated an increase in glucose. Only one experimental medication class, calcium channel blockers, substantially reduced HbA1c and glucose (−0.11%, p = 0.01; and −0.85 mg/dL, p = 0.02, respectively [paired t-test]). The statin and anti-convulsant medication classes were associated with increases in glucose (0.72 mg/dL, p = 2.1 x 10^−7^; and 0.71 mg/dL, p = 5.9e^−4^, respectively [paired t-test]).

### Mendelian randomization

In Table 3, we report our T2D two-sample MR results using S-PrediXcan summary statistics for various indications with putative drug gene targets. In our MR analysis, the strongest evidence for T2D prevention were observed for drugs targeting genes involved in systolic blood pressure, angina, and atrial fibrillation. ACE inhibitors, as proxied by reduced *ACE* gene expression, were predicted to reduce systolic blood pressure by approximately 0.25 mmHg (p = 0.28 [IVW MR association]) per standard deviation decrease in *ACE* expression. Whereas for drugs targeting *KCNJ11* expression (i.e., minoxidil and verapamil), the predicted change in systolic blood pressure was minimal per standard deviation change in *KCNJ11* gene expression. A 16–18% T2D risk reduction was observed for ACE inhibitors (T2D OR = 0.82, 95% CI = 0.78, 0.86, p = 3.3 x 10^−17^ [IVW MR association]) and minoxidil/verapamil (T2D OR = 0.84, 95% CI = 0.81, 0.87, p = 5.0 x 10^−25^ [IVW MR association]) via changes in predicted systolic blood pressure. Reduced atrial fibrillation risk via decreased expression of *HCN3* and *SCN3A* (via HCN channel blockers and sodium channel blockers, respectively) were associated with reduced T2D risk in the MR analysis; however, only sodium channel blockers were observed to reduce T2D risk (T2D OR = 0.25, 95% CI = 0.17, 0.39, p = 4.7 x 10^−11^ [IVW MR association]). Similarly, evidence for T2D risk reduction was also observed for angina risk reduction via verapamil as proxied by reduced *CACNA1A* expression (T2D OR = 0.17, 95% CI = 0.10, 0.29, p = 2.1 x 10^−10^ [IVW MR association]). However, after accounting for potential pleiotropy via changes in correlated indications, only ACE inhibitors demonstrated consistent evidence for a reduction in T2D risk (T2D MVMR OR = 0.86, 95% CI = 0.84, 0.89, p = 4.8 x 10^−06^ [IVW MR association]). The negative control MR (Supplemental Table S7) indicated no bias due to population stratification in the original GWAS studies used for the aforementioned MR analyses proxy long-term effects.

**Table 3:** Summary of estimated mendelian randomization (MR) effects of drug-targeted genetically-predicted gene expression (GPGE) on indications (either continuous or dichotomous) with type 2 diabetes (T2D) risk.

Gene target	Indication-specific GPGE							T2D MR						
	Specific drug or drug class	Drug effect on gene expression	No. of	No. of	Beta^b^	SE	p value	OR^c^	95% CI	p value	Egger	MVMR	95% CI	p value
			SNPs^a^	Tissues^a^							p value^d^	OR^e^		
	**Systolic blood pressure**													
*ACE*	ACE inhibitors	Antagonist	108	11	−0.253	0.223	0.28	0.82	0.78–0.86	3.3 x 10^–17^	0.2	0.86	0.84–0.89	4.8 x 10^–6^
*KCNJ11*	Minoxidil	Agonist	454	13	0.016	0.13	0.9	0.84	0.81–0.87	5.0 x 10^–25^	0.7	n/a	n/a	n/a
*KCNJ11*	Verapamil	Antagonist	454	13	−0.016	0.13	0.9	0.84	0.81–0.87	5.0 x 10^–25^	0.7	n/a	n/a	n/a
*TH*	Metyrosine	Antagonist	29	2	0.061	0.323	0.88	0.79	0.43–1.45	0.45	n/a	n/a	n/a	n/a
*CACNA1A*	Verapamil	Antagonist	1	1	2.032	0.847	1.6 x 10^–2^	0.64	0.56–0.73	2.1 x 10^–10^	n/a	n/a	n/a	n/a
*GCK*	Sitaxentan	Agonist	66	2	0.321	0.187	0.34	0.78	0.71–0.87	2.3 x 10^–6^	n/a	n/a	n/a	n/a
	**Angina**													
*CACNA1A*	Verapamil	Antagonist	1	1	0.511	0.2	1.1 x 10^-2^	0.17	0.10–0.29	2.1 x 10^–10^	n/a	n/a	n/a	n/a
	**Atrial fibrillation**													
*HCN3*	HCN chan. Blockers	Antagonist	114	5	0.102	0.035	4.6 x 10^-2^	0.75	0.53–1.06	0.11	1.4 x 10^–2^	0.92	0.77–1.09	0.43
*SCN3A*	Na + chan. Blockers	Antagonist	83	3	0.041	0.01	5.2 x 10^-2^	0.26	0.17–0.39	4.7 x 10^-11^	0.71	n/a	n/a	n/a
	**Bipolar disorder**													
*SCN3A*	Elpetrigine	Antagonist	46	1	0.15	0.074	4.3x10^-2^	0.92	0.69–1.22	0.55	n/a	n/a	n/a	n/a
	**Rheumatoid arthritis**													
*MAPK3*	Sulindac	Antagonist	95	8	−2.382	0.555	3.6 x 10^–3^	1.01	0.99–1.01	0.08	0.72	n/a	n/a	n/a
	**Glaucoma**													
*BCL2*	Isosorbide	Antagonist	46	1	0.148	0.075	4.9 x 10^–2^	1.01	0.85–1.20	0.93	n/a	n/a	n/a	n/a
	**Congestive heart failure**													
*ACE*	ACE inhibitors	Antagonist	28	1	0.184	0.073	1.1 x 10^–2^	1.31	1.02–1.68	3.2 x 10^–2^	n/a	n/a	n/a	n/a
	**Pain**													
*IKBKE*	Amlexanox	Antagonist	242	8	−0.015	0.003	6.0 x 10^–4^	1.22	0.78–1.88	0.38	0.17	n/a	n/a	n/a
*TAP2*	Olcegepant	Antagonist	259	4	−0.004	0.006	0.6	0.08	0.01–1.36	0.08	0.82	n/a	n/a	n/a
*OPRL1*	Opiod analgesics	Agonist	244	14	0.012	0.002	1.0 x 10^–5^	38.3	18.1–81.2	1.7 x 10^–21^	1.6 x 10^–3^	n/a	n/a	n/a
	**Coronary artery disease**													
*HCN3*	Ivabradine	Antagonist	140	6	−0.076	0.028	4.0 x 10^–2^	1.43	1.06–1.93	2.0 x 10^–2^	0.09	1.26	1.03–1.54	4.9 x 10^–2^
	**Epilepsy**													
*SCN3A*	Anticonvulsants	Antagonist	1	1	0.058	0.028	3.7 x 10^–2^	0.6	0.17–2.08	0.42	n/a	n/a	n/a	n/a

a Number of statistically significant tissues (p value < 0.05) from each indication-specific GPGE model, and the number of SNPs that were used in the GPGE model.

b Summarized Indication-specific GPGE effects using random-effects meta-analysis across the statistically significant tissues.

c Inverse-variance weighted MR analysis for T2D risk using statistically significant tissues from the GPGE model as instruments.

d Egger regression p value < 0.05 indicates the potential for directional pleiotropy.

e Multivariable MR (MVMR) used to estimate associations with T2D risk adjusted for GPGE for other correlated indications/traits. Systolic blood pressure adjusted for congestive heart failure (ACE); atrial fibrillation adjusted for coronary artery disease (HCN3) or bipolar disorder and epilepsy (SCN3A), and coronary artery disease adjusted for atrial fibrillation (HCN3).

## Discussion

Our study has demonstrated an approach for genetics-informed drug repurposing for diabetes by leveraging the power of genomics data, drug annotation databases, EHRs, and contemporary statistical methods for causal inference. A strength of this approach is the rapid and cost-efficient ability to prioritize candidate drugs for clinical trials on the basis of pre-clinical experiments using patient studies and MR.

We demonstrated that calcium-channel blockers are a good target for repurposing for T2D, supported by evidence across all arms of the investigation. This medication demonstrated substantially lower reductions in glycemic indices than the traditional diabetes medications. Our results may be of particular importance to patients with pre-diabetes and hypertension where preferential use of CCBs for blood pressure control may have an additive impact on glycemic control compared with other antihypertensive therapies. We also observed reductions in glycemic indices with another anti-hypertensive medication group, ACE inhibitors. About half of the experimental ACE inhibitors demonstrated significant decreases in both glucose and HbA1c, however, the relative effect sizes varied across sites and depended on the medication type suggesting ACE inhibitor effectiveness to lower glycemic indices depends on type, dose, and possibly the population in which it was used. These findings are similar to clinical trials of ACE inhibitors that have also demonstrated inconsistent results.

The results of this study demonstrate the feasibility and strength of our approach for computational drug repurposing discovery. While many of the potential repurposing medications for glucose reduction have been explored in other trials, we believe these results demonstrate the feasibility of this approach for other disease conditions. Further, because these results derive from two real world clinical populations, they are likely to be robust. These results suggest medications that could be used preferentially to treat concomitant disease while providing potential additional benefit to patients at risk for diabetes or with inadequately controlled diabetes.

This study also demonstrates the importance of careful consideration of study design when performing secondary studies in EHR data sources across sites. We will discuss these considerations in greater detail below. Ascertainment and utilization of pharmacy data in EHRs is a complicated process and it is important to acknowledge limitations in the interpretation of response to prescription start, as medication prescription does not always lead to use. There is also the possibility that a patient is taking other prescription or nonprescription medications that are not reflected in their electronic chart. Another important medication-related limitation is that, unlike the discovery population, the replication population does not have prescription fill data and relied on prescription mention. While we believe the impacts of this limitation are relatively small, as the number of included subjects for most medications in the replication set reflected the ten-fold decrease in sample size we would expect, there were higher prescriptions of prilocaine and lidocaine. The higher prescription of these medications in the replication population may be due to real changes in medication use. It is also possible the increase may reflect an inaccurate capture of prescription, e.g. patients had a second mention of these medications in their record during the six month follow-up period but they were in fact not continuing to take the medication, which would be mitigated by pharmacy fill data. Other potential limitations include the requirement that there be a continuous measurement that can be compared over time, and the relatively short timeframe under observation. An emulated target trial might be a better design for long-term prescriptions in which we may be able to assume that treatment assignment remains consistent throughout the time period under study, or where there are no continuous labs to observe that are related to disease state*.* This potential non-differential exposure misclassification for binary exposures (i.e., treatment vs. non-treatment) will bias results towards the null. However, by additionally incorporating MR in our approach, we hoped to account for this by proxying various medication using GPGE in order to estimate unbiased effects of potential drug-repurposing candidate medications for reducing T2D risk.

Despite these limitations, the self-controlled case series design has many advantages as it allows each individual patient to act as their own control. It is also important to consider that therapeutic response may vary based on patient demographics and health, so effects may be isolated to particular segments of the population. By utilizing a national health care system in the discovery cohort, VA, and a regional healthcare system, VUMC, we had the ability to test response across various environmental regions and demographics. Conveniently, when available this study design allows for further refinement to address specific portions of the study population relating to environment exposures or demographics. The ability to rapidly evaluate acute changes in glycemic indices from two large-scale EHRs provides a real-world impact of the genetically identified medications on patients.

We demonstrate one such refinement analysis with results stratified by EHR-reported race is presented. In this test case we see that the results are largely similar across levels of this variable. Future studies can further develop the experimental strategy to evaluate specific potential confounders and effect modifiers. Data curated for self-controlled case series might also be used for dose–response studies, where dosing is sufficiently variable. Due to inherent limitations in EHRs including data sparsity and unaccounted confounding the current design uses the self-controlled case series to identify acute changes in glycemic indices and Mendelian Randomization to proxy long-term effects of the intervention. Further, the medications identified in the SCCS have established side-effect and they are use in routine medical care therefore the potential for repurposing for diabetes control is strengthened by these established burden considerations. Our approach could be coupled with well-curated clinical cohorts that overcome limitations of EHR systems to allow researchers to consider these long-term effects in a more homogeneous population. Using our design with these cohorts researchers could consider both the short-term effect on the response variables, glycemic indices, as well as the long-term effects such as adverse events and all-cause mortality.

The additional support for a causal effect of the identified drugs provided by the MR analyses provides both epidemiological rigor and evidence of a biological mechanism at work in the phenomenon of the glucose-lowering effect of CCBs.

In conclusion, we present a computational approach to drug repurposing that has the potential to be used for other conditions and with other data resources. We demonstrated this approach with a SCCS design in two large EHR systems and with MR using extant results from large-scale GWAS. We identified CCBs as a candidate treatment for high glucose and observed some evidence for ACE inhibitors as well. This strategy can be implemented for other outcomes with access to sufficient quantities of EHR data and informatics expertise.

## Contributors

M.M.S., K.M.L., J.K., N.K.K., J.H.B., V.M.W, D.R.M, K.R.H., P.D.R, S.L.C., J.L, J.A.L., M.V, and T.L.E. were involved in discussions of study design and provided expert consult for relevant areas. M.M.S., K.M.L, J.K, J.A.L., and N.K.K. were responsible for data access, verification, validation, acquisition, analysis, and presentation of results. M.M.S., K.M.L, J.K, and J.H.B. was responsible for statistical analyses. M.M.S., M.V, T.L.E were responsible for initial manuscript creation. M.M.S., K.M.L., J.K., N.K.K., J.H.B., V.M.W, D.R.M, K.R.H., P.D.R, S.L.C., J.L, J.A.L., M.V, and T.L.E. were involved in the critical review of the manuscript and provided approval of the final manuscript. M.V. and T.L.E. oversaw all project details. The corresponding author attests that all listed authors meet authorship criteria and that no others meeting the criteria have been omitted. All authors read and approved the final manuscript.

## Data sharing statement

Summary level statistical results will be made available upon reasonable request to corresponding authors. Individual level data can be made available following institutional agreements.

## Declaration of interests

J.H.B. declares previous funding from the National Institutes of Health-National Eye Institute. P.D.R. declares a previous institutional grant from Dexcom, Inc for retrospective analysis of VA EHR data on glucose monitoring. J.A.L. has received research grants from Alnylam Pharmaceuticals, Inc, Astellas Pharma, Inc, AstraZeneca Pharmaceuticals LP, Biodesix, Celgene Corporation, Cerner Enviza, GlaxoSmithKline PLC, Janssen Pharmaceuticals, Inc., Kantar Health, Myriad Genetic Laboratories, Inc., Novartis International AG, Parexel International Corporation through the University of Utah or Western Institute for Veteran Research outside the submitted work.
