# Supplementary material for: A genetically supported drug repurposing pipeline for diabetes treatment using electronic health records
Source: eBioMedicine. 2023 Jul 1;94:104674. doi: 10.1016/j.ebiom.2023.104674 (PMC10328805; doi:10.1016/j.ebiom.2023.104674)

GROUP	DRUG	beta (95% CI)	p
Glucose-Increasing	PREDNISONE	2.91 (2.35, 3.46)	<1.0e-08
Glucose-Increasing	PROPRANOLOL	1.64 (1.05, 2.23)	5.8e-08
Glucose-Increasing	CARVEDILOL	2.38 (1.29, 3.47)	1.7e-05
Glucose-Increasing	AMITRIPTYLINE	0.78 (0.28, 1.27)	2.1e-03
Glucose-Increasing	DEXAMETHASONE	7.87 (1.93, 13.81)	9.4e-03
Glucose-Increasing	ATENOLOL	0.38 (0.09, 0.67)	9.5e-03
Glucose-Increasing	HYDROCHLOROTHIAZIDE	1.90 (-0.03, 3.83)	0.054
Glucose-Increasing	BISOPROLOL	2.79 (-1.19, 6.78)	0.17
Glucose-Increasing	METHYLPREDNISOLONE	9.66 (-6.73, 26.05)	0.25
Glucose-Increasing	PAROXETINE	0.34 (-0.24, 0.91)	0.25
Glucose-Increasing	HYDROCORTISONE	3.37 (-3.04, 9.79)	0.30
Glucose-Increasing	NORTRIPTYLINE	0.37 (-0.38, 1.12)	0.33
Glucose-Increasing	METOPROLOL	1.92 (-2.03, 5.88)	0.34
Glucose-Increasing	SERTRALINE	1.54 (-2.42, 5.51)	0.45
Glucose-Increasing	LEVOFLOXACIN	-0.78 (-3.08, 1.52)	0.51
Glucose-Increasing	CITALOPRAM	-0.57 (-2.67, 1.53)	0.59
Glucose-Increasing	FLUOXETINE	-0.09 (-0.46, 0.27)	0.61
Glucose-Increasing	MOXIFLOXACIN	-1.10 (-5.39, 3.20)	0.62
Glucose-Increasing	SOTALOL	0.47 (-1.52, 2.45)	0.65
Glucose-Increasing	RISPERIDONE	-3.33 (-20.48, 13.81)	0.70
Glucose-Increasing	QUETIAPINE	0.11 (-0.51, 0.73)	0.72
Glucose-Increasing	EZETIMIBE	1.03 (-4.82, 6.88)	0.73
Glucose-Increasing	CIPROFLOXACIN	1.13 (-6.98, 9.23)	0.79
Glucose-Increasing	DOXEPIN	0.15 (-1.17, 1.47)	0.82
Glucose-Increasing	NADOLOL	-0.61 (-7.05, 5.84)	0.85
Glucose-Increasing	OLANZAPINE	0.37 (-6.34, 7.09)	0.91
Glucose-Reducing	GLIMEPIRIDE	-18.42 (-25.73, -11.10)	8.0e-07
Glucose-Reducing	PIOGLITAZONE	-9.31 (-14.85, -3.78)	9.7e-04
Glucose-Reducing	LIRAGLUTIDE	-22.83 (-37.78, -7.87)	2.8e-03
Glucose-Reducing	METFORMIN	-24.64 (-43.50, -5.77)	0.010
Glucose-Reducing	GLIPIZIDE	-25.89 (-56.74, 4.96)	0.10
Glucose-Reducing	SITAGLIPTIN	-16.33 (-35.97, 3.31)	0.10
Glucose-Reducing	INSULIN	-17.55 (-41.22, 6.11)	0.15
Glucose-Reducing	GLYBURIDE	-32.14 (-82.14, 17.87)	0.20
Experimental	SIMVASTATIN	0.61 (0.51, 0.72)	<1.0e-08
Experimental	ROSUVASTATIN	1.06 (0.45, 1.67)	6.9e-04
Experimental	TOPIRAMATE	0.96 (0.40, 1.52)	8.4e-04
Experimental	VERAPAMIL	-0.85 (-1.60, -0.11)	0.024
Experimental	FLUVASTATIN	0.88 (0.03, 1.74)	0.043
Experimental	LAMOTRIGINE	0.77 (-0.03, 1.58)	0.059
Experimental	DICLOFENAC	0.36 (-0.05, 0.76)	0.085
Experimental	LIDOCAINE	0.99 (-0.56, 2.54)	0.21
Experimental	LISINOPRIL	0.09 (-0.06, 0.23)	0.23
Experimental	PRAVASTATIN	1.26 (-0.85, 3.38)	0.24
Experimental	ENALAPRIL	-0.59 (-1.61, 0.42)	0.25
Experimental	DABIGATRAN ETEXILATE	0.93 (-0.75, 2.60)	0.28
Experimental	SALMON CALCITONIN	0.84 (-0.68, 2.36)	0.28
Experimental	SULINDAC	0.41 (-0.38, 1.20)	0.31
Experimental	MINOXIDIL	1.61 (-1.93, 5.16)	0.37
Experimental	DOFETILIDE	1.92 (-2.45, 6.30)	0.39
Experimental	ASPIRIN	-0.13 (-0.42, 0.17)	0.40
Experimental	LOVASTATIN	1.28 (-1.74, 4.30)	0.41
Experimental	SILDENAFIL	1.88 (-2.80, 6.57)	0.43
Experimental	DIPYRIDAMOLE	0.98 (-1.85, 3.81)	0.50
Experimental	LEVETIRACETAM	-1.48 (-6.42, 3.47)	0.56
Experimental	OXCARBAZEPINE	-4.31 (-19.38, 10.75)	0.57
Experimental	MICONAZOLE	-1.53 (-6.89, 3.83)	0.58
Experimental	BENAZEPRIL	-0.21 (-1.03, 0.62)	0.62
Experimental	ISOSORBIDE	0.50 (-1.51, 2.50)	0.63
Experimental	PHENYTOIN	0.24 (-0.78, 1.27)	0.64
Experimental	PROPAFENONE	0.91 (-3.00, 4.83)	0.65
Experimental	RAMIPRIL	0.30 (-1.79, 2.39)	0.78
Experimental	ATORVASTATIN	0.29 (-1.92, 2.49)	0.80
Experimental	ISOSORBIDE DINITRATE	-0.20 (-2.02, 1.61)	0.83
Experimental	PRIMIDONE	-0.11 (-1.76, 1.54)	0.90
Experimental	PENTOXIFYLLINE	0.10 (-1.97, 2.17)	0.92
Experimental	FOSINOPRIL	0.02 (-0.44, 0.48)	0.93
Experimental	PREGABALIN	0.08 (-4.37, 4.53)	0.97
Experimental	PRILOCAINE	-0.06 (-3.31, 3.19)	0.97

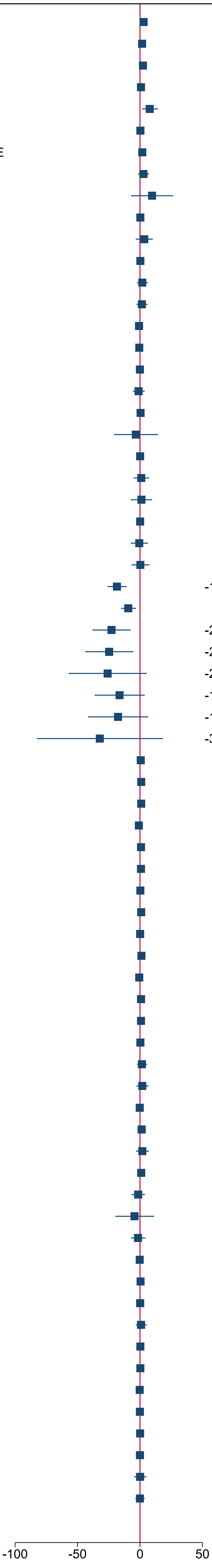

Supplement: Supplemental_Fig. S1 [file mmc1.pdf]
