# Supplementary figures and images for: A genetically supported drug repurposing pipeline for diabetes treatment using electronic health records
Source: eBioMedicine. 2023 Jul 1;94:104674. doi: 10.1016/j.ebiom.2023.104674 (PMC10328805; doi:10.1016/j.ebiom.2023.104674)

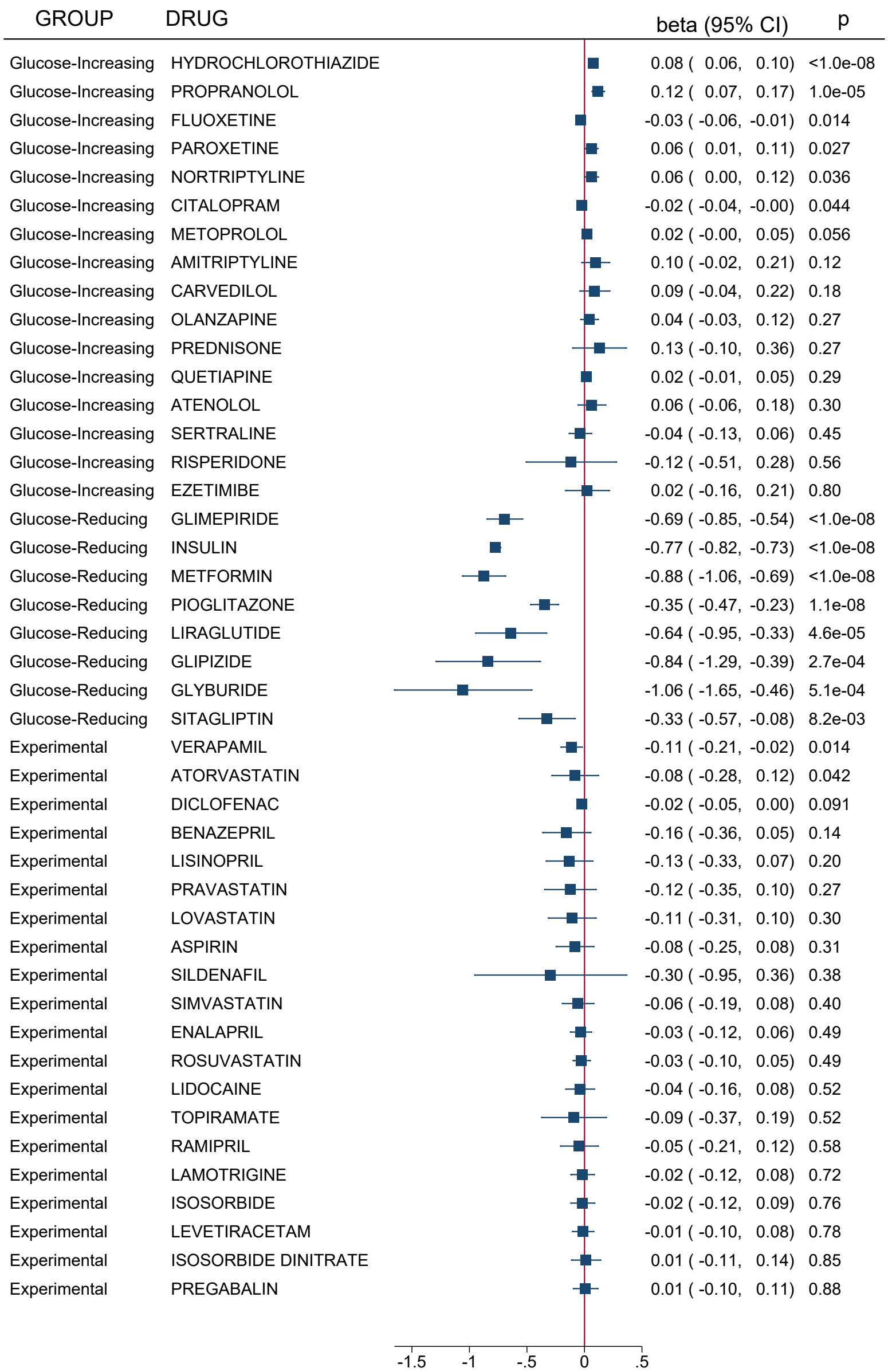

Supplement: Supplemental_Fig. S2 [file mmc2.pdf]
